# piRNA Clusters Need a Minimum Size to Control Transposable Element Invasions

**DOI:** 10.1093/gbe/evaa064

**Published:** 2020-03-27

**Authors:** Robert Kofler

**Affiliations:** Institut für Populationsgenetik, Vetmeduni Vienna, Wien, Austria

**Keywords:** transposable elements, piRNA clusters, population genetics, forward simulations

## Abstract

piRNA clusters are thought to repress transposable element (TE) activity in mammals and invertebrates. Here, we show that a simple population genetics model reveals a constraint on the size of piRNA clusters: The total size of the piRNA clusters of an organism must exceed 0.2% of a genome to repress TE invasions. Moreover, larger piRNA clusters accounting for up to 3% of the genome may be necessary when populations are small, transposition rates are high, and TE insertions are recessive. If piRNA clusters are too small, the load of deleterious TE insertions that accumulate during a TE invasion may drive populations extinct before an effective piRNA-based defense against the TE can be established. Our findings are solely based on three well-supported assumptions: 1) TEs multiply within genomes, 2) TEs are mostly deleterious, and 3) piRNA clusters act as transposon traps, where a single insertion in a cluster silences all TE copies in *trans*. Interestingly, the piRNA clusters of some species meet our observed minimum size requirements, whereas the clusters of other species do not. Species with small piRNA clusters, such as humans and mice, may experience severe fitness reductions during invasions of novel TEs, which is possibly even threatening the persistence of some populations. This work also raises the important question of how piRNA clusters evolve. We propose that the size of piRNA clusters may be at an equilibrium between evolutionary forces that act to expand and contract piRNA clusters.

## Introduction

Transposable elements (TEs) are short stretches of DNA that selfishly propagate within genomes (Doolittle and Sapienza 1980; Orgel and Crick 1980). It is thought that the proliferation of TEs is mostly deleterious to hosts, where negative effects of TEs can arise by three mechanisms: 1) Ectopic recombination among TEs can lead to deleterious chromosomal rearrangements, 2) TE insertions may have direct negative effects, for example, by disrupting genes or regulatory regions, and 3) the products of TEs, such as the *transposase*, can generate deleterious effects (e.g., DNA breaks) (Nuzhdin 1999). Despite this largely selfish activity, some TE insertions may confer beneficial effects on hosts, such as resistance to insecticides (Daborn et al. 2002; González et al. 2008; Casacuberta and González 2013). However, the distribution of the fitness effects of TE insertions remains an important open question (Arkhipova 2018; Kofler 2019).

The unrestrained proliferation of TEs can drive host populations to extinction (Brookfield and Badge 1997; Kofler 2019); thus, the spread of TEs needs to be controlled. The proliferation of TEs may be controlled at the population level, by negative selection against TE insertions, and at the host level, for example, by small RNAs that repress TE activity (Charlesworth and Charlesworth 1983; Charlesworth and Langley 1989; Brennecke et al. 2007; Blumenstiel 2011; Kofler 2019). In mammals and invertebrates, the host defense system relies on piRNAs, small RNAs between 23 and 29 nt in size (Gunawardane et al. 2007; Brennecke et al. 2008). These piRNAs bind to PIWI-clade proteins that direct the repression of TEs at both transcriptional and posttranscriptional levels (Brennecke et al. 2007; Gunawardane et al. 2007; Sienski et al. 2012; Le Thomas et al. 2013). piRNAs are derived from discrete genomic loci, called piRNA clusters, which may make up substantial portions of genomes (Brennecke et al. 2007). In *Drosophila*, for example, piRNA clusters account for 3.5% of the genome (Brennecke et al. 2007).

It is believed that the proliferation of an invading TE is stopped when one copy of the TE jumps into a piRNA cluster, which triggers the production of piRNAs against the TE that ultimately silence all TE copies in *trans* (Bergman et al. 2006; Malone and Hannon 2009; Zanni et al. 2013; Goriaux et al. 2014; Yamanaka et al. 2014; Duc et al. 2019; Ozata et al. 2019). This view is known as the trap model, because piRNA clusters act as genomic traps for active TEs (Bergman et al. 2006). The trap model is currently widely supported by many different observations. For example, artificial sequences inserted into piRNA clusters yield piRNAs complementary to the inserted sequence, and a single insertion in a piRNA cluster is sufficient to silence a reporter construct in *trans* (Josse et al. 2007; Muerdter et al. 2012). Furthermore, a study directly observing TE invasions noted a rapid emergence of piRNA cluster insertions and piRNAs complementary to the invading TE (Kofler et al. 2018). Finally, computer simulations confirmed that piRNA clusters can stop TE invasions (Lu and Clark 2010; Kelleher et al. 2018; Kofler 2019). In particular, large clusters were able to control TE invasions under a wide range of different conditions (Kofler 2019). However, it is unclear if this observation holds for clusters of any size. Here, we tested the hypothesis that piRNA clusters have a minimum size using computer simulations of TE invasions under the trap model. We found that piRNA clusters must account for at least 0.2% of a genome to stop TE invasions over a wide range of parameters. Even larger piRNA clusters may be necessary when populations are small or TE insertions are recessive.

## Results

**Fig. 1.**
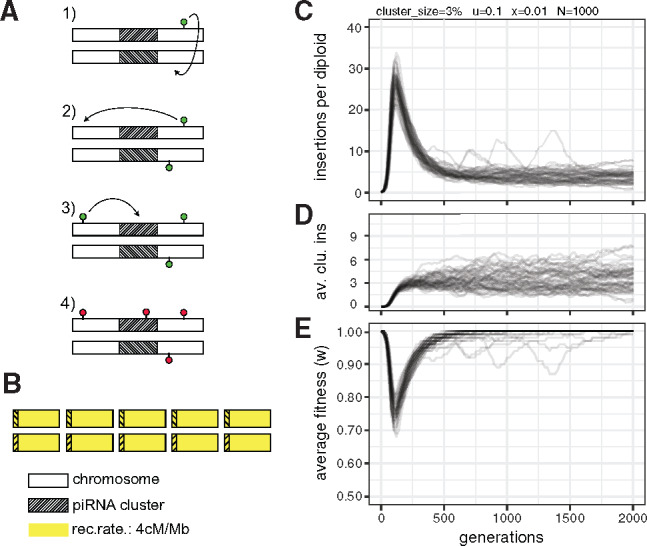
—Dynamics of TE invasions with piRNA clusters and deleterious TE insertions. (*A*) Under the trap model, the proliferation of an active TE (green) is stopped when one copy jumps into a piRNA cluster (i.e., the trap; hatched area), which deactivates all TE copies in *trans* (red). (*B*) We simulated five chromosomes with a size of 10 Mb for a diploid organism. A piRNA cluster was simulated at one end of each chromosome and a recombination rate of 4 cM/Mb was used. (*C*) The abundance of TE insertions during an invasion. Fifty replicates are shown. (*D*) Number of cluster insertions per diploid during an invasion (av. clu. ins). (*E*) Average fitness during an invasion.

We performed forward simulations of TE invasions under the trap model to test the hypothesis that piRNA clusters need to have a minimum size (Bergman et al. 2006). The trap model holds that the proliferation of an invading TE is stopped when a TE copy jumps into a piRNA cluster, which triggers the production of piRNAs that silence all TE copies in *trans* (fig. 1*A*). Accordingly, we assumed that TEs multiply at a transposition rate of *u *>* *0 in individuals without a cluster insertion and at a rate of *u *=* *0 in individuals with a cluster insertion. Excisions were ignored as they have a minor influence on the dynamics of TE invasions with piRNA clusters (Kofler 2019). We simulated diploid organisms, with five chromosomes of size 10 Mb and a recombination rate of 4 cM/Mb (fig. 1*B*). If not mentioned otherwise, the population size was *N *=* *1,000. A dual-strand piRNA cluster was simulated at one end of each chromosome (fig. 1*B*). The size of the piRNA clusters was identical for all individuals within a population. We measured the size of piRNA clusters as a percentage of the genome, as the relative size of piRNA clusters (as a percent), not the absolute size (in bp), determines the invasion dynamics of TEs (Kofler 2019; Kelleher et al. 2018). In our simulations, piRNA clusters with a size of 1% correspond to one cluster of 100 kb at one end of each chromosome (fig. 1*B*). Initially, we assumed that each TE reduces the fitness of the host (*w*) by a constant factor (*x*) such that w=1−xn, where *n* is the number of TE insertions per diploid (Charlesworth and Charlesworth 1983). Furthermore, we assumed that TE insertions in piRNA clusters incur no negative fitness effects (i.e., *x *=* *0 for cluster insertions). To avoid the stochastic early stages of an invasion, where TEs may get lost due to genetic drift (Le Rouzic and Capy 2005; Kofler 2019), we launched each TE invasion by randomly distributing 1,000 insertions in individuals of the initial population. These insertions have an initial population frequency of 1/2N.

Invasions of deleterious TEs under the trap model show a characteristic pattern (fig. 1*C*–*E*; with *u *=* *0.1, *x *=* *0.01, and a cluster size of 3%; see also Kelleher et al. 2018). Initially, the TE rapidly multiplies within the genome, which markedly reduces the fitness of the host (fig. 1*C* and *E*). Next, individuals accumulate increasing numbers of cluster insertions, which slow down the spread of the TE (fig. 1*C* and *D*). Finally, negative selection removes deleterious TE insertions, which restores the fitness of the host to nearly initial levels (fig. 1*C* and *E*). In our example, the TE invasion temporarily reduced host fitness by ∼20–30% (fig. 1*E*). In these simulations, piRNA clusters accounted for 3% of the genome. The size of the piRNA clusters is a major factor determining the number of TEs that accumulate during an invasion, that is, more TE insertions will accumulate when piRNA clusters are small (Kofler 2019; Kelleher et al. 2018). The fitness reduction during a TE invasion will be more dramatic for smaller piRNA clusters (Kelleher et al. 2018). We surmised that populations may go extinct when piRNA clusters are very small.

**Fig. 2.**
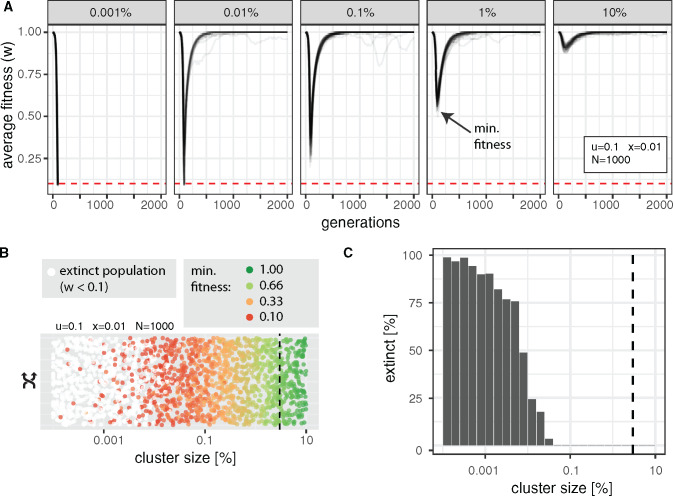
—piRNA clusters need a minimum size to control TE invasions. (*A*) Average fitness of populations during TE invasions with different sizes of piRNA clusters (top panel). Fifty replicates are shown. We refer to the lowest fitness during an invasion as the “minimum fitness” (arrow). Red dashed lines indicate the extinction threshold (*w *<* *0.1). With a cluster size of 0.001%, all populations went extinct. (*B*) Influence of the cluster size on the extinction of populations. Each dot represents the result of a single simulation with a randomly drawn cluster size. For nonextinct populations, the minimum fitness is shown. The size of piRNA clusters in *Drosophila melanogaster* is indicated as a dashed black line (3%). Random numbers are used for the *y*-axis. (*C*) Histogram showing the fraction of extinct populations for different cluster sizes. No extinct populations were found for clusters >0.1%.

To test these hypotheses, we simulated TE invasions with cluster sizes ranging from 0.001% to 10% (fig. 2*A*). Throughout this work, we refer to the lowest fitness of a population during an invasion as the minimum fitness (fig. 2*A*). Furthermore, we assumed that a population went extinct if its average fitness dropped below 0.1 (i.e., min. fitness < 0.1). With an average fitness of 0.1, ∼16–23% of the individuals in a population have a fitness of zero (assuming a linear fitness function; the effect of nonlinear fitness functions will be explored later). These individuals will not contribute any offspring to the next generation. We introduced the extinction threshold of 0.1 to avoid unrealistic simulation conditions where, for example, very few survivors with fitness just above zero could reconstitute the entire population of the next generation.

As expected, the minimum fitness was close to 1.0 for large clusters (fig. 2*A*; 10% cluster size). However, the minimum fitness dropped substantially with decreasing cluster sizes (fig. 2*A*). For clusters with a size of 0.001%, the minimum fitness dropped below 0.1 for all simulated populations, that is, all populations went extinct. This demonstrates that transposon traps, such as piRNA clusters, need a minimum size to prevent the extinction of populations. To identify the required size of piRNA clusters, we performed 2,000 simulations with randomly chosen cluster sizes (fig. 2*B*). As expected, for clusters with a size of 0.001% the vast majority of the populations went extinct (fig. 2*B* and *C*). No extinct populations were observed for clusters >0.1% (fig. 2*B* and *D*). Hence, in the simulated scenario, the minimum size of piRNA clusters was ∼0.1%.

Thus far, we have assumed that cluster insertions have no deleterious fitness effects. However, it is possible that TE insertions in piRNA clusters reduce host fitness. For example, cluster insertions will increase genome size, and a large genome may be deleterious (Petrov 2001). We found that negative selection on cluster insertions only had a minor influence on the minimum fitness during an invasion, and consequently, on the extinction rate of populations (supplementary fig. S1, Supplementary Material online). The minimum size of piRNA clusters was slightly larger when all TE insertions, including cluster insertions, had negative fitness effects (*x *=* *0.01; supplementary fig. S1 and table S1, Supplementary Material online). For the remainder of the manuscript, we assumed that cluster insertions have no fitness cost to the host (i.e., *x *=* *0.0 for cluster insertions).

**Fig. 3.**
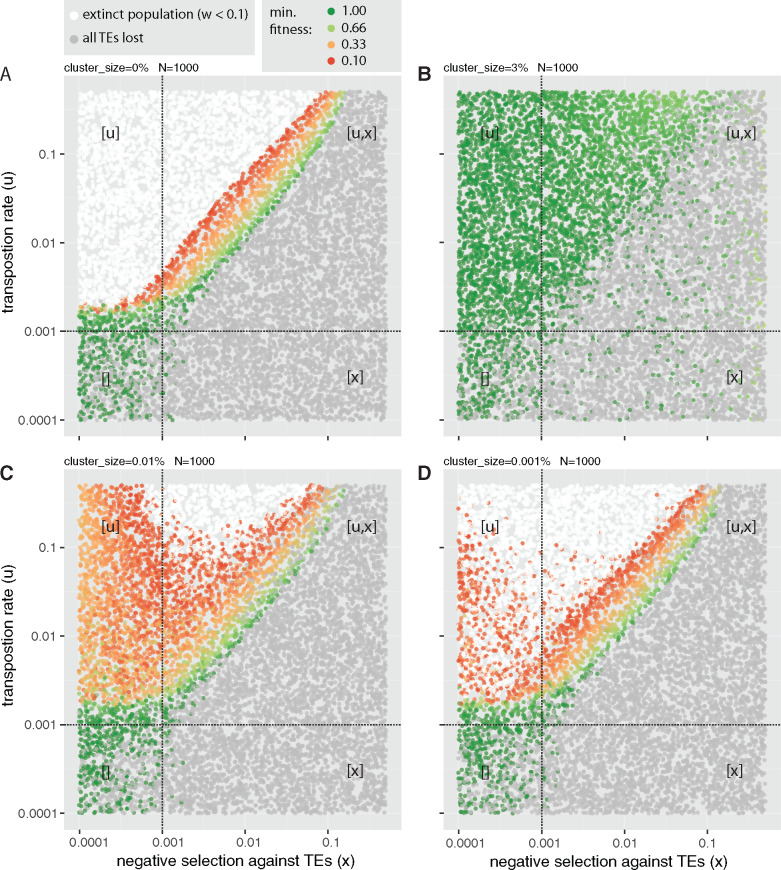
—Small piRNA clusters offer insufficient protection from the extinction of host populations. Each dot represents the outcome of a single simulated TE invasion. The transposition rate (*u*) and negative effect of TEs (*x*) were randomly selected. (*A*) In the absence of piRNA clusters, extinct populations are observed when u≫x. (*B*) Extinct populations are not observed when piRNA clusters are large (3% of the genome). (*C*) For small piRNA clusters (0.01%), extinct populations are observed when transposition rates are high and negative effects are intermediate. (*D*) Extinct populations are common for very small piRNA clusters (0.001%). Depending on the efficacy of negative selection and transposition (N*u>1 and N*x>1 with *N *=* *1,000), the parameter space can be divided into four quadrants. Factors that are effective in a quadrant are shown in brackets.

To identify regions of the parameter space where populations with small piRNA clusters are vulnerable to extinction, we performed simulations with randomly chosen transposition rates (*u*) and negative effects of TEs (*x*). We followed the invasions for 5,000 generations and recorded the results (fig. 3). In the absence of piRNA clusters, three principal outcomes can be observed (Kofler 2019). First, populations can go extinct when u≫x (fig. 3*A*). Second, populations may lose all TE insertions when negative selection against TEs is strong (*x *>* u*; fig. 3*A*). The minimum fitness of these populations is usually close to the maximum fitness (*w*_max_ = 1.0) (supplementary fig. S2, Supplementary Material online). Third, stable TE copy numbers may be attained when the number of TEs removed by negative selection equals the number of novel TE insertions gained by transposition (transposition selection balance) (Charlesworth and Charlesworth 1983; Charlesworth and Langley 1989). In the absence of piRNA clusters, stable TE copy numbers are solely observed in a narrow region of the parameter space (fig. 3*A*; assuming a linear fitness function; the window is larger with nonlinear fitness functions [Kofler 2019]). If large piRNA clusters (3%) are introduced into the model, the extinction of populations is prevented over the entire parameter space (fig. 3*B*; see also Kofler 2019). Furthermore, stable TE copy numbers are observed for a wide range of parameters (*u *>* x*; fig. 3*B*). However, when piRNA clusters are small (0.01%), extinct populations reemerge (fig. 3*C* and *D*). Extinct populations are mainly observed when transposition rates are high and negative effects of TEs are intermediate (fig. 3*C*). This raises the question: Why do intermediate deleterious effects of TEs lead to extinctions when piRNA clusters are small? Strongly deleterious TEs are usually quickly removed by negative selection. These TEs are thus unable to accumulate copy numbers high enough to drive populations to extinction. Weakly deleterious TEs may accumulate high copy numbers before cluster insertions stop an invasion. However, the cumulative deleterious effect of TEs with weak effects may be insufficient to drive populations to extinction. Only TEs with intermediate effects can accumulate high enough numbers and be sufficiently deleterious to drive a population to extinction.

**Fig. 4.**
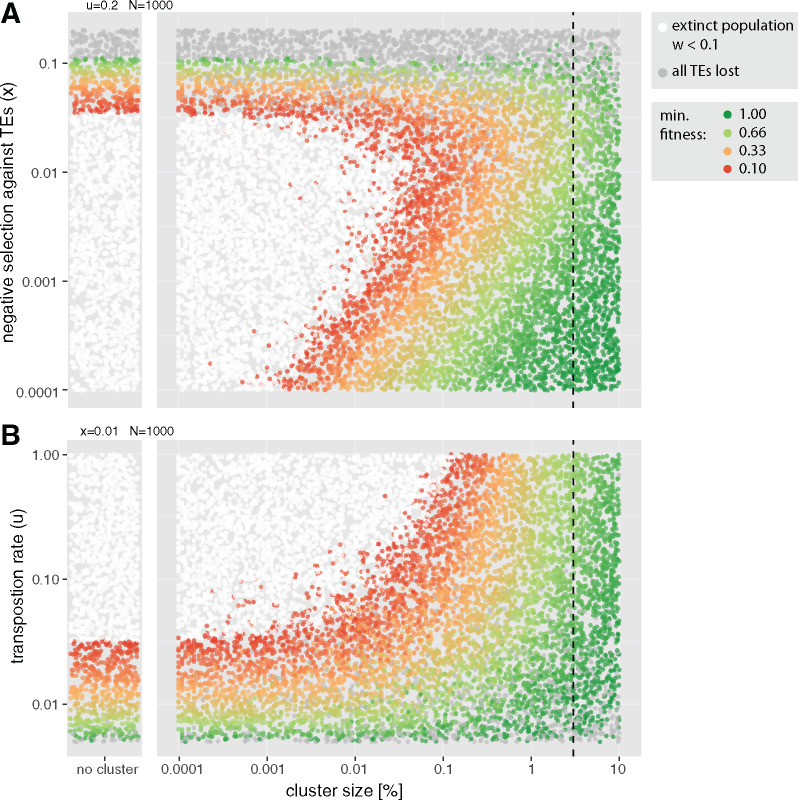
—The influence of the negative effects of TEs (*A*) and transposition rates (*B*) on the minimum size of piRNA clusters. Each dot represents the outcome of a single simulation. The cluster size, the transposition rate, and the negative effect of TEs were randomly selected. To identify regions of the parameter space where piRNA clusters are not necessary to control TEs (nonwhite dots in the left panel), we show results for simulations performed without piRNA clusters. Dashed lines indicate the size of piRNA clusters in *Drosophila melanogaster* (3%). Large piRNA clusters with sizes of 0.1–1% are necessary to control TEs when transposition rates are high and negative effects of TEs are intermediate.

Next, we aimed to identify the minimum size of piRNA clusters under different scenarios. We first investigated the influence of the transposition rate (*u*) and the negative effect of TEs (*x*) using >20,000 simulations with randomly chosen parameters (fig. 4). As TE insertions are usually rapidly purged from populations when *x *>* u* (fig. 3), we performed simulations mostly with transposition rates larger than the negative effects of TEs (fig. 4). We identified regions of the parameter space that do not require piRNA clusters for controlling TEs using simulations performed without piRNA clusters (fig. 4*A* and *B*; nonwhite dots in left panels). piRNA clusters had a minimum size whenever piRNA clusters were necessary to control TE invasions (fig. 4*A* and *B*). The largest piRNA clusters were necessary when the negative effects of TEs were intermediate (fig. 4*A*) and the transposition rates were high (fig. 4*B*). The largest piRNA clusters of an extinct population had a size of 0.16%. However, this extinction was observed for a simulation with a high transposition rate of *u *=* *0.96. So far, the largest observed transposition rates were slightly smaller, with u=0.2−0.6 (Robillard et al. 2016; Kofler et al. 2018).

**Fig. 5.**
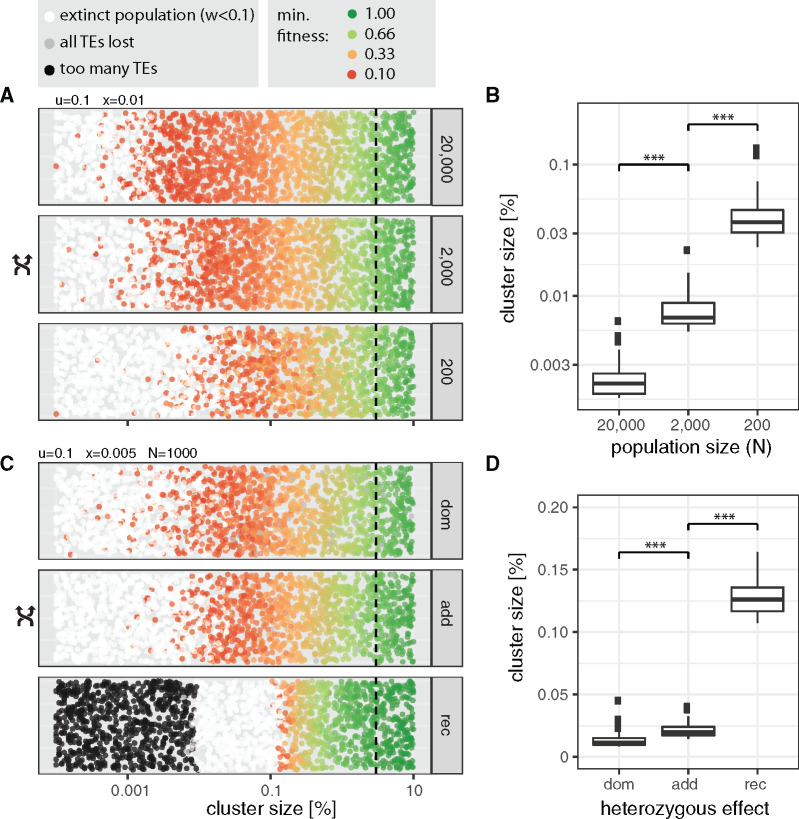
—Influence of the population size (*A*, *B*) and the heterozygous effect (*C*, *D*) on the minimum size of piRNA clusters. (*A*, *C*) Each dot represents the outcome of a single simulation with randomly chosen cluster sizes, population sizes (200−20,000), or heterozygous effects of TEs (dom dominant, add additive, rec recessive). Dashed lines indicate the size of piRNA clusters in *Drosophila melanogaster* (3%). Random numbers are used for the *y*-axis. (*B*, *D*) The 50 largest clusters of extinct populations. Significance was estimated with Wilcoxon rank-sum tests. *** *P *<* *0.001.

As the efficacy of negative selection, an important factor counteracting TEs, depends on population sizes, we investigated the influence of this factor (Gillespie 2010). We performed 2,000 simulations with randomly chosen cluster sizes for three different population sizes (20,000, 2,000, and 200; fig. 5*A*). The population size had a significant influence on the minimum size of piRNA clusters, where small populations required the largest piRNA clusters (fig. 5*B*; for effect sizes see supplementary table S1, Supplementary Material online). With the smallest evaluated population size (*N *=* *200), the largest cluster of an extinct population had a size of 0.13%.

Thus far, we have assumed that TE insertions have additive fitness effects, such that homozygous TE insertions reduce host fitness twice as much as heterozygous insertions. However, it is feasible that TE insertions have recessive deleterious effects (Charlesworth and Langley 1989; Lee and Langley 2010). For example, TE insertions may disrupt genes for which a single intact copy is sufficient to generate the wild type (haplosufficient). On the other hand, it is feasible that TE insertions are dominant, for example, due to *trans*-epigenetic effects (Lee et al. 2020). To investigate the impact of heterozygous effects on the minimum cluster size, we used the fitness function $w=1-2xi_{\mathrm{hom}}-2hxi_{\mathrm{het}}$, where *i*_hom_ and *i*_het_ are the number of homozygous and heterozygous insertion sites, respectively. For the additive case (*h *=* *0.5), this equation simplifies to our standard fitness formula, w=1−xn, as the number of TE insertions per diploid genome is $n=2i_{\mathrm{hom}}+i_{\mathrm{het}}$. This approach allows us to model dominant (*h *=* *1.0), additive (*h *=* *0.5), and recessive (*h *=* *0.0) effects of TE insertions. During some simulations with recessive effects, the number of accumulated TE insertions exceeded our computational resources. We terminated these simulations (>25,000 insertions per diploid; fig. 5*C*, black dots). However, as this problem solely occurred for very small piRNA clusters, an order of magnitude smaller than the clusters where extinctions were first observed, this limitation will not influence our conclusions (fig. 5*C*). The heterozygous effect had a significant influence on the minimum size of piRNA clusters, where the largest clusters were required for recessive insertions and the smallest for dominant insertions (fig. 5*D*; for effect sizes, see supplementary table S1, Supplementary Material online). With recessive insertions, the largest cluster of an extinct population had a size of 0.16%, and with dominant insertions, a size of 0.04%. Our findings highlight the influence of the efficacy of negative selection against TEs on the minimum size of piRNA clusters. When the efficacy of negative selection is high (large populations or dominant insertions), small clusters are sufficient to control TEs, whereas large clusters are necessary when the efficacy of negative selection is weak (small populations or recessive insertions).

Thus far, we have assumed that all TE insertions reduce host fitness by the same amount, irrespective of the genomic insertion site. However, it is likely that different TE insertions have very diverse fitness effects (Pasyukova et al. 2004). For example, insertions into coding sequences are probably more harmful than insertions into intergenic regions. We evaluated the effect of heterogeneous fitness effects of TE insertions using the fitness function: $w=1-\sum_{i=1}^{n}x_{i}$. This equation simplifies to our standard fitness formula (w=1−xn) when all TE insertions have identical effects. We mixed TE insertions with strong (*x *=* *0.1), moderate (*x *=* *0.01), weak (*x *=* *0.001), and very weak (*x *=* *0.0001) deleterious effects at different proportions (supplementary fig. S3*A*, Supplementary Material online). The heterogeneity of the effect sizes had a significant influence on the minimum size of piRNA clusters (Kruskal–Wallis rank-sum test with the 50 largest clusters of extinct populations; $\chi^{2}=235,\;P<2.2e-16$; supplementary fig. S3*B*, Supplementary Material online). In all simulated scenarios, piRNA clusters had a minimum size, ranging from 0.005% to 0.05% (supplementary fig. S3*B*, Supplementary Material online).

**Fig. 6.**
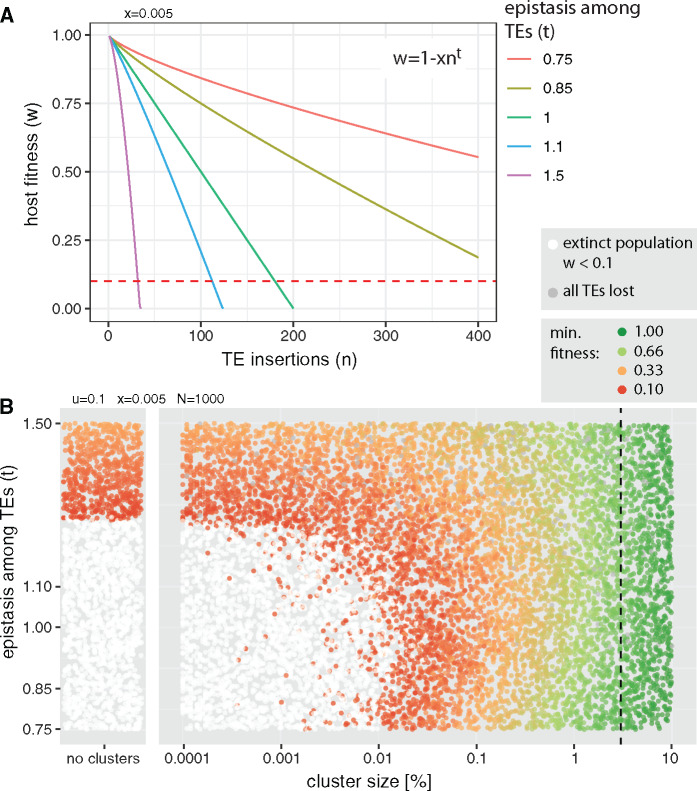
—Influence of epistatic interactions (*t*) among TEs on the minimum size of piRNA clusters. (*A*) The fitness of the host may decrease linearly (*t *=* *1), steeply (*t *>* *1), or slowly (*t *<* *1) with the TE copy number. The red dashed line indicates the extinction threshold (*w *<* *0.1). (*B*) Parameter space with epistatic TE interactions. Each dot represents the outcome of a single simulation with a random cluster size and an epistatic interaction term (*t*). Results of simulations performed without piRNA clusters are shown to identify regions of the parameter space where piRNA clusters are not necessary to control TEs (nonwhite dots in the left panel). The dashed line indicates the size of piRNA clusters in *Drosophila melanogaster* (3%).

Up to this point, we have assumed that each novel TE insertion reduces host fitness by the same amount, irrespective of the number of TE copies already present in the host genome. Hence, we assumed that the host fitness declines linearly with the TE copy number. Nevertheless, it is feasible that host fitness decreases more steeply with the TE copy number (Charlesworth and Charlesworth 1983). For example, ectopic recombination among distant TEs could lead to highly deleterious genomic rearrangements (e.g., inversions or translocations), and the number of ectopic recombination events may increase exponentially with TE abundance (Charlesworth and Charlesworth 1983; Charlesworth and Langley 1989; Montgomery et al. 1991). On the other hand, it is also conceivable that the decline of host fitness slows down with TE copy number. For example, the products of a few TE insertions may suffice to reduce host fitness to some extent, but the products of further TE copies may only have a minor impact. Following Charlesworth and Charlesworth (1983), we modeled interactions among TEs using the equation $w=1-xn^{t}$. This formula allows us to model the linear (*t *=* *1; yielding our standard fitness formula w=1−xn), steep (*t *>* *1), and slow (*t *<* *1) decrease of host fitness with TE copy number (fig. 6*A*). We performed 10,000 simulations with randomly chosen cluster sizes and interaction terms ranging from 0.75 (slow decline) to 1.5 (steep decline; fig. 6). In the simulated scenario, piRNA clusters were not necessary to control TE invasions when *t *>* *1.25 (nonwhite dots; fig. 6*B*). This is in agreement with previous work showing that a steep fitness decline (*t *>* *1) extends the parameter space over which TEs may be controlled in the absence of piRNA clusters (Charlesworth and Charlesworth 1983; Kofler 2019). Nevertheless, large piRNA clusters can by themselves protect populations from extinction over the entire parameter space (Kofler 2019). However, at less steep fitness declines (*t *<* *1.25), extinct populations were observed for small piRNA clusters. Interestingly, the minimum size of piRNA clusters only varied moderately among the different epistatic interactions of TEs (0.017% with t≈0.75; 0.045% with t≈1.0; 0.015% with t≈1.2; ANOVA with the twenty largest clusters of extinct populations P<3.2e−14). Our finding that piRNA clusters have a minimum size is thus robust and applies to different nonlinear declines of host fitness with TE copy number.

**Fig. 7.**
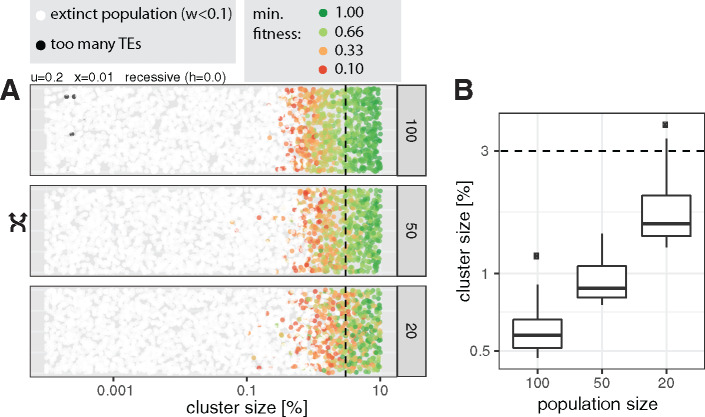
—The minimum size of piRNA clusters under the worst-case scenario, that is, recessive TE insertions of intermediate effects, high transposition rates, and three different small population sizes (right panel). (*A*) Influence of cluster size on the extinction of populations. Each dot represents the result of a single simulation with a randomly drawn cluster size. The minimum fitness is shown for nonextinct populations. The size of piRNA clusters in *Drosophila melanogaster* is shown as a dashed black line (3%). Random numbers are used for the *y*-axis. (*B*) The 50 largest piRNA clusters of extinct populations.

Under the previously simulated scenarios, piRNA clusters with a size of 0.2% were sufficient to protect populations from extinction. However, the joint effect of some important parameters may necessitate even larger clusters. We thus investigated the minimum size of piRNA clusters during a worst-case scenario, that is, 1) small population size (20–100), 2) recessive TE insertions, 3) high transposition rates (*u *=* *0.2), and 4) intermediate negative effects of TEs (*x *=* *0.01; fig. 7). In these scenarios, piRNA clusters need a size of 1–3% to protect populations from extinction (fig. 7; for effect sizes see supplementary table S1, Supplementary Material online).

In summary, we conclude that piRNA clusters need a size of at least 0.2% to protect populations from extinction over a wide range of parameters. In a worst-case scenario involving small populations and recessive insertions, clusters accounting for up to 3% of the genome may be necessary to control TEs.

## Discussion

Here, we used a simple population genetics model to reveal a constraint on the size of transposon traps, such as piRNA clusters. To control TE invasions over a wide range of parameters, piRNA clusters need to have a minimum size of 0.2% of the genome. When clusters are smaller, populations may go extinct from the load of deleterious TE insertions that accumulate during a TE invasion.

Our finding that piRNA clusters have a minimum size relies on three well-supported assumptions. First, TEs amplify within genomes at a certain rate. Second, as TE insertions are mostly deleterious, host fitness decreases with TE copy number such that hosts will eventually die from the cumulative effects of the TE insertions. Third, piRNA clusters act as transposon traps such that a TE insertion in a piRNA cluster represses all other copies of the TE in *trans*.

There is little doubt about our assumption that TEs multiply within host genomes (Wicker et al. 2007; Burt and Trivers 2008). However, we also rely on the widely used assumption that the transposition rate is constant during an invasion (in the absence of piRNAs) (e.g., Charlesworth and Charlesworth 1983; Charlesworth 1991; Lu and Clark 2010; Kelleher et al. 2018; Kofler 2019). It is not entirely clear whether this assumption holds, as very few studies estimated transposition rates during TE invasions. Kofler et al. (2018) traced *P-element* invasions in experimental *Drosophila simulans* populations for 40–60 generations. Before the TE was silenced by piRNAs, the *P-element* had a rather uniform transposition rate of about *u *=* *0.15 in hot conditions and *u *=* *0.05 in cold conditions (Kofler et al. 2018). Hence, a constant transposition rate is currently a reasonable assumption.

Our assumption that TE insertions are mostly deleterious is also well supported (Yukuhiro et al. 1985; Mackay 1989; Mackay et al. 1991; Houle and Nuzhdin 2004; Pasyukova et al. 2004; Blumenstiel et al. 2014). Moreover, it seems highly unlikely that a complex host defense mechanism, such as the piRNA pathway, could have evolved if TEs were not deleterious. Nevertheless, several major questions remain. Most importantly, the distribution of the fitness effects of TE insertions and the rate at which fitness decays with TE copy number remain unclear (Arkhipova 2018; Kofler 2019). However, our finding that piRNA clusters need a minimum size to control TE invasions is robust and applies under different assumptions about the deleterious effects of TEs. Our conclusion would not hold under a model where host fitness never approaches zero, even when an organism accumulates vast amounts of TEs, but such a model seems unlikely. Indeed, there is ample support for our assumption that TEs may cause the death (or sterility) of hosts. The well-described hybrid dysgenesis systems are good examples (Kidwell et al. 1977). As the piRNA-mediated defense against TEs is maternally transmitted, TEs are reactivated in offspring resulting from crosses of males with a TE and females without the same TE (Brennecke et al. 2008). This TE reactivation may lead to diverse defects. The *I-element* causes developmental defects that prevent the hatching of larvae (Bucheton et al. 1976; Wang et al. 2018). *Hobo* and *P-element* activity lead to dysgenic and mostly sterile ovaries (Kidwell et al. 1977; Kidwell 1983; Blackman et al. 1987; Hill et al. 2016). Sterile gonads may also result from reactivation of multiple TE families, as observed in a hybrid dysgenesis syndrome in *Drosophila virilis* (Evgen'ev et al. 1997; Erwin et al. 2015). Furthermore, individuals with defects in components of the piRNA pathway, such as *Aub* or *Piwi*, are frequently infertile (Lin and Spradling 1997; Ma et al. 2009; Czech et al. 2013; Théron et al. 2018). This infertility may, at least partially, be due to a high TE activity in individuals without functional TE defense (Ma et al. 2009).

Finally, our assumption that piRNA clusters act as traps that control TE activity is well supported. First, it was observed that a single *P-element* insertion in a piRNA cluster is sufficient to repress a reporter construct in *trans* (Josse et al. 2007). Second, the insertion of an artificial sequence into a piRNA cluster triggered the production of piRNAs complementary to the sequence (Muerdter et al. 2012). Third, the transposons ZAM and Idefix are inactive in strains with an insertion of these two TEs in the somatic piRNA cluster flamenco, but active in strains without these cluster insertions (Zanni et al. 2013). Fourth, a de novo insertion of ZAM into a germline piRNA cluster restores the repression of ZAM (Duc et al. 2019). Fifth, piRNA clusters are frequently composed of TE sequences (Brennecke et al. 2007), supporting the view that clusters carry the trapped remnants of past TE invasions (but see pachytene clusters in mice [Girard et al. 2006; Ernst et al. 2017]). Finally, computer simulations confirm that genomic traps, such as piRNA clusters, may stop TE invasions (Lu and Clark 2010; Kelleher et al. 2018; Kofler 2019).

The minimum size of piRNA clusters identified in this work (0.2%) rests on the further assumption that TE insertion sites are random. Some transposons, however, have pronounced insertion preferences (Sultana et al. 2017) that may lead to over or underrepresentation of TE insertions within piRNA clusters. It has also been surmised that piRNA clusters can somehow attract TE insertions (Brennecke et al. 2007). Our estimates of the minimum size of piRNA clusters will be too small if TEs avoid inserting into piRNA clusters and too large if TEs preferentially jump into piRNA clusters (or if piRNA clusters attract TEs). As insertion biases vary among TE families (Linheiro and Bergman 2012; Jakšić et al. 2017), robust protection from extinction may require piRNA clusters larger than the estimated 0.2%.

Furthermore, we simulated dual-strand clusters where TE insertions of any orientation generate piRNAs that silence TEs (Malone et al. 2009). For uni-strand clusters, which silence somatic TEs, cluster insertions need to be antisense (Malone et al. 2009). As this basically halves the probability of obtaining a piRNA-producing cluster insertion, we expect that uni-strand clusters need to be larger than dual-strand clusters.

Our estimated minimum size of piRNA clusters is based solely on a population genetics model. It is likely that biochemical processes also constrain the size of piRNA clusters. For example, de Vanssay et al. (2012) showed that loci with seven tandem copies of a transgene may be converted into a piRNA cluster by maternally inherited small RNAs, whereas loci with fewer tandem copies could not be converted.

Our finding that piRNA clusters need a minimum size of 0.2% (3% in the worst case) to control TE invasions raises the important question of whether clusters of different species actually meet this requirement. The size of germline clusters in *Drosophila melanogaster* is ∼3% of the genome (Brennecke et al. 2007). These clusters thus provide comprehensive protection from TEs, even under our worst-case scenario. Nevertheless, a TE with a strong insertion bias against piRNA clusters could overcome this defense and drive populations to extinction. In *D. melanogaster*, a distinct set of transposons is active in the somatic tissue surrounding the germline (Song et al. 1997; Blumenstiel 2011; Barckmann et al. 2018). These TEs are controlled by a single somatic cluster, flamenco (Li et al. 2009; Malone et al. 2009), which has a size of ∼0.15% (assuming a flamenco size of 300 kb and a genome size of 200 Mb; Brennecke personal communication; Bosco et al. 2007). As another example, in Koala, piRNA clusters account for 0.17% of the genome (Yu et al. 2019). Somatic clusters in *D. melanogaster* and piRNA clusters in Koala provide sufficient protection under the majority of the simulated scenarios. However, under a worst-case scenario, that is, small populations and recessive TE insertions, these clusters may be too small to control TEs. In mice, humans, and rats, pachytene piRNA clusters account for ∼0.1% of the genome (Girard et al. 2006). These clusters likely offer insufficient protection from TE invasions under multiple simulated scenarios. Nevertheless, pachytene piRNA clusters may be able to stop TE invasions if the TEs have an insertion bias into these clusters (Ernst et al. 2017). Alternatively, some organisms may have multiple layers of defense against TEs. For example, Kruppel-associated box zinc-finger proteins (KRAB-ZFPs) may silence TEs in some mammals (Yang et al. 2017). KRAB-ZFPs bind to TE sequences and induce heterochromatin formation (Yang et al. 2017). However, some TEs continue to be active even when targeted by KRAB-ZFPs, and KRAB-ZFPs only target some TE families (Yang et al. 2017). Furthermore, it not clear if KRAB-ZFPs are mostly responsible for the long-term maintenance of TE repression, or if they also silence newly invading TEs. However, it is difficult to see how the sequence specificity of KRAB-ZFPs, which is determined by tandem arrays of C2H2 zinc fingers (Yang et al. 2017), could rapidly adapt to the sequence of a newly invading TE. Thus, in contrast to the piRNA pathway, which provides a generalized defense mechanism capable of trapping and silencing any mobile element, KRAB-ZFPs likely constitute a more specialized defense layer, requiring adaptation of the DNA binding sites to the sequence of each novel TE. It would be interesting to estimate the size of piRNA clusters in more species. Blatant violations of our minimum size requirements may indicate that the piRNA pathway is not the primary defense against TEs in some organisms or that the simple version of the trap model, which presumes that a TE invasion is controlled by random TE insertions into piRNA clusters, is incomplete. For example, it may be necessary to incorporate paramutations into the trap model (de Vanssay et al. 2012). Under this scenario, maternally transmitted piRNAs may convert TE insertions into piRNA-producing loci (Le Thomas et al. 2014). Silencing of a novel TE invasion with paramutations could thus proceed like a chain reaction wherein increasing amounts of TE insertions in different individuals are converted into piRNA-producing loci. The source of the first piRNAs that trigger this chain reaction may again be a random insertion into piRNA clusters. In contrast to the simple trap model, where several cluster insertions are required in each individual (Kofler 2019), cluster insertions are only expected in very few individuals under a trap model with paramutations (because euchromatic TE insertions may also yield piRNAs [see also Mohn et al. 2014; Shpiz et al. 2014]). The minimum size of piRNA clusters would thus be much reduced. Currently, many questions about paramutations remain to be answered: 1) Are paramutations important for silencing novel TE invasions, or mostly for maintaining the silencing of a TE?; 2) which fraction of the TE insertions could potentially be converted into piRNA-producing loci?; 3) are paramutated loci stably inherited over generations?; and 4) what is the role of the environment in paramutations (e.g., Casier et al. 2019)?

Finally, it is also feasible that in some organisms, piRNAs against a newly invading TE emerge independently of cluster insertions. For example, it was proposed that the emergence of piRNAs against the KoRV virus in koala is triggered by unspliced TE transcripts (Yu et al. 2019). The mechanism responsible for recognizing unspliced TE transcripts is, however, still unclear. Furthermore, it is not clear if this process silences DNA transposons and how this mechanism distinguishes between transcripts derived from TEs and genes.

Our work also raises the important question of which evolutionary forces shape the evolution of piRNA clusters. In our simulations, populations with small piRNA clusters went extinct whereas populations with large clusters survived. However, it is not necessary to invoke such group selection arguments (Maynard Smith 1964, 1976) to explain the evolution of piRNA clusters in natural populations. The size of piRNA clusters could be polymorphic in natural populations, such that individuals with small and large clusters can be found within a single population. If individuals with large clusters end up with fewer deleterious TE insertions than individuals with small clusters, large clusters may be positively selected. This hypothesis is supported by recent simulation studies, which found that TE insertions within piRNA clusters may be positively selected (Lu and Clark 2010; Kelleher et al. 2018; Kofler 2019). Large clusters will on average end up with more TE insertions than small clusters. Due to the perfect linkage between piRNA clusters and its TE insertions, large clusters will be positively selected. The size of piRNA clusters may thus grow to a level where extinctions of populations are no longer expected.

On the other hand, evolutionary forces may exist that restrict the size of piRNA clusters. If the TE defense machinery comes at a cost to the host (Koonin et al. 2020), large piRNA clusters may be costlier to maintain than small clusters, especially when novel TE invasions are rare. The fitness cost of piRNA clusters may stem from ectopic recombination among cluster insertions or the cellular resources expended for generating vast amounts of piRNAs derived from large portions of genomes. This raises the intriguing possibility that the size of piRNA clusters is at an equilibrium between evolutionary forces that act to expand and contract them. If this hypothesis is true, natural populations should exhibit variations in the size of piRNA clusters. Such variations could exist. A recent study found that the subtelomeric piRNA cluster X-TAS is present in most wild strains of *D. melanogaster* but is frequently lost in lab strains (Asif-Laidin et al. 2017). Future studies may shed light on the variation of piRNA clusters in natural populations.

## Materials and Methods

### Simulation Software

All simulations were performed with the Java tool Invade (Kofler 2019). Invade performs individual-based forward simulations of TE activity in diploid organisms. For this work, we released a version (0.8.07) that implements the following new features: 1) computes the minimum fitness during an invasion, 2) offers support for heterozygous effects of TE insertions, and 3) offers support for mixed deleterious fitness effects of TE insertions. In each generation, Invade performs the following steps in the given order: 1) Mate pairs are formed based on the fitness of the individuals, 2) haploid gametes are generated based on the recombination map, 3) novel TE insertions are introduced into the gametes of parents without an insertion in a piRNA cluster, 4) zygotes are formed, 5) the fitness and the number of cluster insertions in the new individuals are computed, and 6) the output is generated (optional).

### Simulated Scenarios

We simulated a genome with five chromosomes of 10 Mb (–genome Mb:10,10,10,10,10) and a recombination rate of 4 cM/Mb (–rr cM_Mb:4,4,4,4,4). Invasions were launched by randomly introducing 1,000 TE insertions in individuals of the initial population (–basepop seg:1000). Simulations under our worst-case scenario were launched by randomly distributing 100 insertions in the starting population. This was necessary because 1,000 insertions with an *x *=* *0.01 in a small population of 20 reduce the fitness of individuals in the starting population by 50% (with 100 insertions, fitness is only reduced by 5%). The transposition rate (e.g., –u 0.1), the negative effect of TE insertions (e.g., –x 0.01), the population size (e.g., –N 1000), and the cluster size (e.g., –cluster kb:each:100) varied among simulated scenarios. The key parameters used for each scenario are shown at the top of the figures. All TE invasions were simulated for 5,000 generations (–gen 5000), except for the scenario in which we varied the transposition rate from 0.005 to 1.0, in which 10,000 generations were used. To cover the parameter space in one or two dimensions, we used Python scripts that launched between 2,000 and 12,500 simulations with randomly chosen parameter combinations.

Different rates of fitness decay with increasing TE copy numbers were simulated with an epistatic interaction term (e.g., –t 1.0; with the fitness function $w=1-xn^{t}$). To simulate dominant, recessive, and additive TE effects, a heterozygous effect was provided (recessive –nsmodel het:0.0; additive –nsmodel het:0.5; dominant –nsmodel het:1.0). Mixed deleterious effects of TE insertions were simulated by providing the effect sizes of the insertion sites (–nsmodel site:0.1,0.001). Equal amounts of sites were simulated for each effect size.

All statistical analyses were performed using R (R Core Team 2019), and visualizations were done with the ggplot2 library (Wickham 2016).

The sizes of piRNA clusters of humans, mice, and rats were computed using the data of Girard et al. (2006) (supplementary tables S2–S4, Supplementary Material online). We summed up the length of each cluster and divided this sum by the approximate genome size of 3,000 Mb (cluster sizes: mouse 2.70 Mb, human 3.16 Mb, rat 3.29 Mb).

## Supplementary Material

evaa064_Supplementary_Data

## Notes

**Data deposition:** Invade is available at https://sourceforge.net/projects/invade/. All Python and R scripts used for the analysis are available at SourceForge https://sourceforge.net/projects/te-tools/ (folder “minsize”).
